# Contrast analysis for competing hypotheses: A tutorial using the R package cofad

**DOI:** 10.3758/s13428-025-02833-w

**Published:** 2025-10-29

**Authors:** Mirka Henninger, Simone Malejka, Johannes Titz

**Affiliations:** 1 Faculty of Psychology, University of Basel, Basel, Switzerland; 2 Department of Psychology, University of Cologne, Cologne, Germany; 3 Institute of Psychology, Chemnitz University of Technology, Chemnitz, Germany

**Keywords:** Contrast analysis, Competing contrasts, Multi-group analysis, Hypothesis testing, Experimental research

## Abstract

Researchers in psychology traditionally use analysis of variance to examine differences between multiple groups or conditions. A less well-known, but valuable alternative is contrast analysis — a simple statistical method for testing directional, theoretically motivated hypotheses that are defined prior to data collection. In this article, we review the core concepts of contrast analysis for testing hypotheses in between-subjects and within-subjects designs. We also outline and demonstrate the largely unknown possibility of directly testing two competing contrasts against each other. In the tutorial part of the article, we show how such competing-contrast analyses can be conducted in the free, open-source software R using the package cofad. Because competing-contrast analysis is a straightforward, flexible, highly powered, and hypothesis-driven approach, it is a valuable tool to extend the understanding of cognitive and behavioral processes in psychological research.

Psychological researchers traditionally analyze multi-group data using analysis of variance (ANOVA). ANOVAs allow researchers to test omnibus hypotheses about main and interaction effects of (quasi-)experimental factors. A less commonly used alternative is contrast analysis, which can be used to test specific, directional, a priori hypotheses about a specific pattern of group or condition means. This procedure yields several advantages, such as a higher statistical power when the predicted mean pattern is observed in empirical data. Furthermore, contrast analysis provides researchers with the possibility to directly compare two competing hypotheses.

To illustrate the value and the procedure of contrast analyses, we introduce an experiment by Maraver et al. (2021) investigating a false-memory effect as an example from cognitive psychology. The term *false memory* subsumes various phenomena in memory psychology relating to erroneously remembering an event or detail that the person believes to be true, but that did not actually happen or occurred differently than remembered Bernstein et al. (2018). One such memory error is remembering events that were implied or could be inferred from a sentence, but were not explicitly stated (Brewer, 1977). For example, after reading the sentence “The karate champion hit the cinder block” participants may recall that the karate champion broke the cinder block, even though the original sentence did not mention whether the block actually broke.

In their Experiment 1, Maraver et al. (2021) tested whether instructions to imagine the study material can protect against false memories. Participants were presented with everyday action sentences that could induce pragmatic inferences. The encoding instructions were to either *imagine* , to *memorize* , or to *pay attention* to the sentences. Finally, participants were asked to fill in the critical words in a sentence-completion task (e.g., ”The karate champion $\_\_\_\_\_\_$ the cinder block“). Memory performance was measured as the proportion of correctly completed sentences. The condition of interest was the *imagine* instruction, whereas the *memorize* and *pay attention* instructions served as control conditions.^1^

Maraver et al. (2021) expected that imaginal encoding protects against false memories, because generating images improves memory (imagination facilitation; Foley et al., 2006), whereas the two control conditions should not affect memory performance. This hypothesis could be formalized as the following predicted mean pattern $\mu _{\textit{imagine} } > \mu _{\textit{memorize} } = \mu _{\textit{pay attention} }$.

As an alternative to the research question investigated by Maraver et al. (2021), one could formulate a hypothesis regarding the control conditions that may suggest different forms of learning when participants are asked to *memorize* versus to *pay attention* to the sentences. When explicitly instructed to memorize, participants expect a memory test and thus intentionally encode the sentences (Bereiter & Scardamalia, 1989). This in turn could motivate them to select encoding strategies that involve deeper cognitive processing according to the levels-of-processing theory (Craik & Lockhart, 1972). Deeper processing (e.g., thinking about meaning) leads to better memory than shallow processing (i.e., attending to surface features). Hence, the competing hypothesis could be formalized as $\mu _{\textit{imagine} }> \mu _{\textit{memorize} } > \mu _{\textit{pay attention} }$.

In the study conducted by Maraver et al. (2021), a $1\times 3$ between-subjects ANOVA showed significant main effects of the encoding instructions on the proportion of correct responses, $F(2,117) = 28.89, p <.01, \eta _p^2 =.33$. This result indicates that the type of encoding instructions plays a role in memory performance. The post-hoc Bonferroni adjusted comparisons reported by Maraver et al. (2021) showed better memory performance in the *imagine* condition than in the *memorize* or the *pay attention* condition.

## Omnibus versus specific hypothesis tests

As many researchers in experimental psychology, Maraver et al. (2021) used ANOVA and post-hoc tests to analyze their data. In ANOVA, the null hypothesis states that the means in all conditions are equal. This null hypothesis is tested against the alternative hypothesis, stating that at least two conditions have different means. Unfortunately, ANOVAs do not allow researchers to test specific differences between conditions, and post-hoc pairwise comparisons often lack sufficient power. We demonstrate in this tutorial article how specific hypotheses can be tested using contrast analysis and how researchers can assess which of two competing hypotheses is more strongly supported by the data (Steiger, 2004).

Many textbooks emphasize that contrast analysis can serve as a substitute for traditional ANOVA (e.g., Saville & Wood, 1991; Draper & Smith, 1998). For instance, a $2 \times 2$ factorial ANOVA can be reformulated using a set of orthogonal contrasts representing the two main effects and their interaction. Analogously to ANOVA, this approach accounts for the total between-group variance with the advantage of conducting directional tests for each factor and their interaction^2^ (see Appendix A for the contrast weights). Similarly, in one-factor designs with more than two conditions, contrast analysis enables the testing of specific directional hypotheses regarding the expected pattern of condition means. For example, Helmert contrasts can be used to test whether the mean in a control group is lower than the average of two intervention groups, and whether one intervention group outperforms the other (see Appendix A for different sets of contrast vectors). Finally, a single contrast can be used to test whether the observed group means covary with a pattern predicted from theory. In such cases, beyond the general advantages of contrast analysis, this approach avoids a potential type-I error inflation in multiple testing by relying on a single, theory-based test, and offers greater statistical power than post-hoc tests when the observed means align with the hypothesized pattern (Furr, 2008; Langenberg et al., 2023).

We would like to note that contrast analysis is not new. In fact, it has already been promoted in the 1980s and 1990s (e.g., Abelson & Prentice, 1997; Rosenthal & Rosnow, 1985), and is statistically simple and, in principle, can be conducted by hand. Even though some tutorial-style articles are available (e.g., Furr, 2008; Haans, 2018) and contrast analysis is a regular topic in psychological curricula (Sternkopf et al., 2025), the full potential of contrast analysis has not yet been exploited in psychological research (but see de Melo & Terada, 2020; Lachner et al., 2019; Vorauer et al., 2020, for recent applications).

One of the potentials of contrast analysis lies in the possibility to directly test two competing hypotheses against each other (Rosenthal et al., 2000). While competing hypotheses involving only two conditions can be compared in a two-sample or paired *t*-test, testing competing hypotheses across more than two conditions, as in the study by Maraver et al. (2021), is more challenging. Herein, we focus on this potential of contrast analysis and demonstrate how researchers can compare competing contrasts to determine whether one favored hypothesis aligns more closely with the observed data compared to a rival hypothesis. We refer to this procedure as *competing-contrast analysis*.

**Fig. 1:**
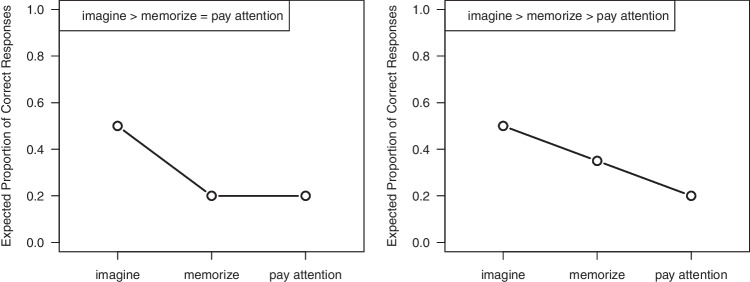
Visualization of the expected proportion of correct responses in the experiment by Maraver et al. (2021). Note. *Left panel*: Visualization of Hypothesis 1. The expected proportion of correct responses is highest in the *imagine* condition and the *memorize* as well as the *pay attention* are proper baseline conditions. *Right panel*: Visualization of Hypothesis 2. The expected proportion of correct responses is highest in the *imagine* condition; the *memorize* condition outperforms the *pay attention* condition because it induces a deeper level of processing at encoding. The expected proportions of correct responses are selected based on performances in a typical memory experiment and this relative pattern of condition means helps to determine the contrast weights

This tutorial is organized as follows. We first review the principles of standard contrast analysis for multi-group comparisons with a priori hypotheses using an example with one experimental factor and three conditions. We then demonstrate the largely unknown advantage of contrast analysis to directly compare two competing hypotheses about the expected mean pattern across these conditions. Finally, using data by Maraver et al. (2021) and Akan et al. (2018), we show how researchers can conduct contrast analyses for independent (between-subjects designs) and dependent samples (within-subjects designs; repeated measurements), and competing hypotheses by hand and using the R package cofad (Titz & Burkhardt, 2021, 2024). An accompanying R script is available on the Open Science Framework (OSF) at https://osf.io/ny5b6/. Throughout the article, we use a nominal $\alpha$-level of $5\%$.

## Contrast analysis

The main idea behind contrast analysis is that a specific, predicted mean pattern across conditions (e.g., based on an a priori hypothesis) is tested against the observed mean pattern across conditions in empirical data. This may include main and interaction effects as in standard ANOVA, but with directional statistical tests, as well as more specific hypotheses. Depending on the type of hypothesis to be tested, researchers have to specify so-called contrast weights for one or multiple contrasts. Researchers can then examine whether the specified contrast weights covary with the mean pattern observed in the data (Rosenthal et al., 2000; Maxwell et al., 2004; Sedlmeier & Renkewitz, 2008). The contrast weights can be directly determined from the to-be-tested hypothesis as described next.

### Determining contrast weights

The contrast vector $\boldsymbol{\lambda }$ of one contrast is composed of *K* contrast weights, one for each experimental condition *i* with $i \in (1,...,K)$. Apart from that, the only formal requirement for contrast weights is that they sum to zero for a given contrast:

1 $$\begin{aligned} \sum _{i = 1}^K\lambda _{i} = 0. \end{aligned}$$

The contrast vector should reflect the relative predicted mean pattern in the *K* groups. Sedlmeier and Renkewitz (2008) propose to determine the contrast weights as follows:

1. derive the relative predicted mean pattern for all experimental conditions from the theory,
2. subtract the average of the expected group means from the mean pattern, and
3. optionally multiply each contrast weight by a constant to obtain more manageable and interpretable values.

These three steps may not sound very intuitive when researchers first come into contact with contrast analysis. At the same time, deriving contrast weights from one’s hypothesis may be a major strength of this statistical method. It encourages researchers to think about the predicted mean pattern at an early stage of the research project. In the light of open science practices and preregistered reports, specifying the expected results as detailed as possible can improve the quality of psychological research (see Lakens, 2019).

**Table 1:** Illustration of how to determine the contrast weights for the predicted mean pattern of Hypothesis 1 ($\mu _{\textit{imagine} } > \mu _{\textit{memorize} } = \mu _{\textit{pay attention} }$) based on the experiment by Maraver et al. (2021)

	*imagine*	*memorize*	*pay attention*
Predicted mean pattern	0.5	0.2	0.2
Subtract average	$0.5-0.3 = 0.2$	$0.2 - 0.3 = -0.1$	$0.2 - 0.3 = -0.1$
Multiply by constant	$0.2 \cdot 5 = 1.0$	$-0.1 \cdot 5 = -0.5$	$-0.1\cdot 5 = -0.5$

Note. The expected mean values are based on the visualization in Fig. 1

**Table 2:** Contrast weights specified for Hypothesis 1 and Hypothesis 2 derived for the experiment by Maraver et al. (2021)

	*imagine*	*memorize*	*pay attention*
	$\lambda _1$	$\lambda _2$	$\lambda _3$
Hypothesis 1: $\mu _{\textit{imagine} } > \mu _{\textit{memorize} } = \mu _{\textit{pay attention} }$	1	$-0.5$	$-0.5$
Hypothesis 2: $\mu _{\textit{imagine} }> \mu _{\textit{memorize} } > \mu _{\textit{pay attention} }$	1	0	$-1$

One way to determine the contrast weights for a given hypothesis is to visualize the expected group means. Each predicted mean pattern can then be translated into a set of contrast weights. Using the study by Maraver et al. (2021) as an example, two contrasting hypotheses can be derived (e.g., Thapar & McDermott, 2001): While imaginal encoding is always expected to protect against false memories and thus should lead to the best memory performance, two different hypotheses for the control conditions can be formulated. On the one hand, the *memorize* and the *pay attention* instruction can be understood as proper control conditions, leading to the same memory performance as illustrated in the left panel of Fig. 1 (Hypothesis 1). On the other hand, according to levels-of-processing theory, *memorize* instructions should lead to deeper encoding and thus better memory performance than *pay attention* instructions as illustrated in the right panel of Fig. 1 (Hypothesis 2).

Once the predicted mean pattern (i.e., the relative differences between the group means) is established, the researcher can determine the contrast weights that reflect this mean pattern and that meet the requirement that the sum of the contrast weights of each contrast is equal to zero. Table 1 illustrates this procedure. For Hypothesis 1, the average of the expected group means is subtracted from the predicted mean pattern and then the result is multiplied by 5 for convenience, to obtain the following vector of contrast weights: $\boldsymbol{\lambda }_{\text {Hyp1}} = (1.0, -0.5,-0.5)$. We can now use these contrast weights to test whether postulating this specific mean pattern makes a good prediction on observed data. Under the null hypothesis, the contrast weights are unrelated to the observed data. Under the alternative hypothesis, the contrast weights covary with the group means in the observed data.

Note that only the relative pattern of contrast weights, not the absolute value of the contrast weights, plays a role in the significance test of the contrast. Some researchers recommend using integer values as weights, others recommend values between $-1$ and $+1$ because the contrast weights reflect the relative weighting of the observed means. In our example, we use the latter approach in which *imagine* is contrasted with the *memorize* and *pay attention* conditions. This is indicated by the absolute weight of 0.5 (i.e., “half” or “average”) for the *memorize* and the *pay attention* condition.

In contrast analysis, researchers often use multiple contrast vectors to test distinct components of a single, a priori hypothesis about the pattern of condition means. In the example by Maraver et al. (2021) with three experimental conditions, the contrast vector $\boldsymbol{\lambda }_{\text {Hyp}1} = (1, -0.5, -0.5)$ derived above tests whether the mean of the first group differs from the average of the second and third group means. Additionally, a second contrast vector $\boldsymbol{\lambda }_{\text {Hyp}2} = (0, 1, -1)$ can be used to test whether the second and third group means differ. Together, these two contrast vectors form a Helmert contrast set, which exhausts the between-group degrees of freedom ($K - 1$ for *K* groups) by specifying two orthogonal contrasts. This analytic strategy — first testing whether the first group differs from the remaining groups and then examining whether the remaining groups differ from one another — is commonly used in contrast analysis (Draper & Smith, 1998; Kaltenbach, 2021; Saville & Wood, 1991). For further details and examples of contrast sets, see Appendix A.

In this tutorial, we pursue a different focus in contrast analysis: We aim to compare competing hypotheses directly by evaluating which of two predicted mean patterns *aligns more closely* – in terms of their covariation – with the observed mean pattern in the data, which we refer to as competing-contrast analysis. This approach evaluates whether both predicted mean patterns covary equally with the observed mean pattern, or whether the observed pattern shows a stronger covariance with the prediction from Hypothesis 1 than with the prediction from Hypothesis 2. In other words, competing-contrast-analysis tests the statistical null hypothesis that the predicted mean pattern based on Hypothesis 1 covaries with the observed mean pattern to the same extent as the predicted mean pattern based on the rival Hypothesis 2. Therefore, it can be understood as a test of theory-based differences between the two predicted mean patterns (Rosenthal et al., 2000; Sedlmeier & Renkewitz, 2008).

### Competing-contrast analysis

Imagine a researcher wants to challenge the hypothesis that $\mu _{\textit{imagine} } > \mu _{\textit{memorize} } = \mu _{\textit{pay attention} }$ and favors the rival hypothesis that $\mu _{\textit{imagine} }> \mu _{\textit{memorize} } > \mu _{\textit{pay attention} }$ wherein the memorizing instructions should lead to deeper processing and is thus more beneficial to memory than paying attention. The resulting contrast weight vector for the second hypothesis is $\boldsymbol{\lambda }_{\text {Hyp2}} = (1,0,-1)$. Table 2 summarizes the contrast vectors for both competing hypotheses.

When using competing-contrast analysis, the contrast vectors representing two competing hypotheses can be directly compared. This is done by calculating the difference between the two contrast vectors. Before computing this difference, the contrast weights must be standardized, such that the resulting weights do not unfairly favor the contrast with higher absolute weights. The result is a new contrast vector describing the standardized difference between the original vectors. The standardized contrast vector $\lambda _i^*$ is given by:

2 $$\begin{aligned} \lambda _{i}^* = \frac{\lambda _i}{s_{\lambda }} \end{aligned}$$

with $s_{\lambda }$ being the standard deviation of the contrast weights, which is defined as

3 $$\begin{aligned} s_{\lambda } = \sqrt{\frac{\sum _{i=1}^K\lambda _i^2}{K}}. \end{aligned}$$

The difference between the two standardized contrast vectors $\lambda _{i,\text {Hyp1}}^*$ and $\lambda _{i,\text {Hyp2}}^*$ can then be used as the contrast vector to test whether Hypothesis 1 fits the data better than Hypothesis 2 :

4 $$\begin{aligned} \lambda _{i,\text {difference}} = \lambda _{i,\text {Hyp1}}^* - \lambda _{i,\text {Hyp2}}^* . \end{aligned}$$

More specifically, if we want to compare the two competing hypotheses in the experiment by Maraver et al. (2021), we first have to standardize their contrast weights depicted in Table 2. The standard deviation of the contrast weights in each contrast is given by:

5 $$\begin{aligned} s_{\lambda _{\text {Hyp1}}} = \sqrt{\frac{1^2 + (-0.5) ^2 + (-0.5)^2 }{3}} = \sqrt{0.5} \end{aligned}$$

and

6 $$\begin{aligned} s_{\lambda _{\text {Hyp2}}} = \sqrt{\frac{1^2 + 0 ^2 + (-1)^2 }{3}} = \sqrt{0.667}. \end{aligned}$$

The standardized contrast weights are:

7 $$\begin{aligned} \boldsymbol{\lambda }_{\text {Hyp1}}^* = \Big ( \frac{1}{\sqrt{0.5}}, \frac{-0.5}{\sqrt{0.5}}, \frac{-0.5}{\sqrt{0.5}} \Big ) = ( 1.41, -0.71, -0.71 ) \end{aligned}$$

and

8 $$\begin{aligned} \boldsymbol{\lambda }_{\text {Hyp2}}^* \!=\! \Big ( \frac{1}{\sqrt{0.667}}, \frac{0}{\sqrt{0.667}}, \frac{-1}{\sqrt{0.667}} \Big ) \!=\! ( 1.22, 0, -1.22). \end{aligned}$$

The resulting contrast vector to test the two competing hypotheses is obtained by subtracting the contrast vectors for each hypothesis as follows:

9 $$\begin{aligned} \boldsymbol{\lambda }_{\text {difference}} = \boldsymbol{\lambda }_{\text {Hyp1}}^* - \boldsymbol{\lambda }_{\text {Hyp2}}^* = ( 0.19, -0.71, 0.51 ). \end{aligned}$$

If a directional test of this contrast is significant, it indicates that Hypothesis 1 corresponds more closely to the observed mean pattern than Hypothesis 2. In other words, we can test theoretically derived differences between two predicted mean patterns to assess whether the observed mean pattern covaries more strongly with Hypothesis 1 compared to Hypothesis 2.

## Contrast analysis for independent samples

Contrast analysis for independent samples (between-subjects designs) can be conducted using the *F*-statistic or the *t*-statistic. Both variants are demonstrated below. The *F*-statistic of contrast analysis is closely related to the *F*-statistic in ANOVAs and may therefore be intuitive for researchers familiar with ANOVA. Note that the degrees of freedom in the numerator of the *F*-value for one contrast are $$ df =1$$, such that $$F = t^2$$ or $$\sqrt{F} = |t|$$. Thus, the *t*-test can conveniently be used instead of the *F*-test, with the advantage of allowing directional (one-tailed) tests. Also note that contrast analysis for $$K=2$$ independent groups (e.g., $$\lambda _1 = -1$$; $$\lambda _2 = 1$$) is formally equivalent to a *t*-test for independent samples (Fitts, 2010; Sedlmeier & Renkewitz, 2008; Maxwell et al., 2004; Wahlsten, 1991; Steiger, 2004).

### Statistical tests

#### *F*-test

Researchers familiar with ANOVA may find contrast analysis easily accessible due to their similarities. At its core, the premise remains unchanged: It is evaluated whether two ways of estimating the population variance yield similar outcomes, which corresponds to an *F*-value close to 1 under the null hypothesis. However, contrast analysis diverges from ANOVA in how it estimates the population variance for the numerator. Because $\sum _{i = 1}^K\lambda _{i} = 0$, the population variance can be written as follows (see Rosenthal et al., 2000):

10 $$\begin{aligned} \hat{\sigma }_{\text {contrast}}^2= \frac{\big (\sum _{i=1}^K\lambda _i\bar{x}_i\big )^2}{\sum _{i=1}^K\frac{\lambda _i^2}{n_i}} \end{aligned}$$

where $n_i$ is the sample size in group *i*.

For the case that $\boldsymbol{\lambda }$ and $\boldsymbol{\bar{x}}$ are independent (as expected under the null hypothesis), $\hat{\sigma }_{\text {contrast}}^2$ provides a reliable estimate for the population variance. However, if they are related in the manner the researcher hypothesizes (as expected under the alternative hypothesis), $\hat{\sigma }_{\text {contrast}}^2$ will be substantially larger. This stems from the fact that $\sum _{i=1}^K\lambda _i\bar{x}_i$ represents the covariation between $\boldsymbol{\lambda }$ and $\boldsymbol{\bar{x}}$ (Rosenthal et al., 2000; Maxwell et al., 2004; Sedlmeier & Renkewitz, 2008).^3^ When $\boldsymbol{\lambda }$ and $\boldsymbol{\bar{x}}$ covary, $\hat{\sigma }_{\text {contrast}}^2$ increases, which in turn leads to an increase in the *F*-statistic:

11 $$\begin{aligned} F = \frac{\hat{\sigma }_{\text {contrast}}^2}{\hat{\sigma }_{\text {within}}^2} \end{aligned}$$

where $\hat{\sigma }_{\text {within}}^2$ represents the mean squared error (MSE) within the groups, serving as an alternative method to estimate the population variance:

12 $$\begin{aligned} \hat{\sigma }^2_{\text {within}} = \frac{\sum _{i = 1}^K\hat{\sigma }_i^2}{K}. \end{aligned}$$

The more accurately the researcher can predict $\boldsymbol{\bar{x}}$ through $\boldsymbol{\lambda }$, the higher both $\hat{\sigma }_{\text {contrast}}^2$ and *F*-value will become. The logic behind ANOVA is retained in contrast analysis, and its results can be presented in a typical ANOVA table. It is important to note that a scenario, in which $\boldsymbol{\lambda }$ covary negatively with $\boldsymbol{\bar{x}}$, would also result in an *F*-value larger than 1. To evaluate the direction of the covariation between $\boldsymbol{\lambda }$ and $\boldsymbol{\bar{x}}$, and to explicitly conduct a directional test with higher power, the *t*-statistic can be used.

#### *t*-test

In order to obtain the test statistic for a *t*-test, we compute the contrast estimate *L*, defined as the sum of the observed means weighted by $\lambda _i$:

13 $$\begin{aligned} L = \sum _{i=1}^K\lambda _i\bar{x}_i. \end{aligned}$$

The *L*-value indicates whether the predicted mean pattern, as described by $\boldsymbol{\lambda }$, covaries with the observed mean pattern $\boldsymbol{\bar{x}}$ in the dataset.

To test whether the contrast estimate significantly differs from zero, a *t*-test with $N-K$ degrees of freedom can be conducted, where *N* represents the total sample size across all groups. The test statistic is then calculated as follows:

14 $$\begin{aligned} t = \frac{L}{\hat{\sigma }_{L}} \end{aligned}$$

with

15 $$\begin{aligned} \hat{\sigma }_{L} = \sqrt{\hat{\sigma }^2_{\text {within}}\sum _{i=1}^K\frac{\lambda ^2_i}{n_i}}. \end{aligned}$$

This closely corresponds to the equation for the *F*-statistic for contrast analysis mentioned above.

#### Example of contrast analysis for independent samples

In the following, we reanalyze the data by Maraver et al. (2021) using contrast analysis with a *t*-statistic. Table 3 shows the contrast weights, means, variances, and sample sizes for the three experimental groups.

**Table 3:** Contrast weights of Hypothesis 1, means and variances of the memory performance variable, as well as sample sizes in the three experimental groups in the experiment reported by Maraver et al. (2021)

	*imagine*	*memorize*	*pay attention*
$\lambda _{\text {Hyp1}}$	1	$-0.5$	$-0.5$
$\bar{x}_i$	0.414	0.200	0.250
$\hat{\sigma }^2_i$	0.025	0.012	0.015
$n_i$	40	40	40

*Note:* Means and variances are rounded to three decimals

To test Hypothesis 1, the contrast estimate and resulting empirical *t*-value are computed as follows:

16 $$\begin{aligned} L_{\text {Hyp1}}\approx &  1 \cdot 0.414 + (-0.5) \cdot 0.2 + (-0.5) \cdot 0.25 \approx 0.189,\nonumber \\ \hat{\sigma }^2_{\text {within}}\approx &  \frac{0.025 + 0.012 + 0.015}{3} \approx 0.017,\nonumber \\ \hat{\sigma }_{L_{\text {Hyp1}}}= &  \sqrt{\hat{\sigma }^2_{\text {within}}\sum _{i=1}^K\frac{\lambda ^2_i}{n_i}}\nonumber \\\approx &  \sqrt{0.017\Big ( \frac{1^2}{40} + \frac{(-0.5)^2}{40} + \frac{(-0.5)^2}{40} \Big )}\nonumber \\\approx &  \sqrt{0.017 \cdot 0.038} \approx 0.025, \end{aligned}$$

and

17 $$\begin{aligned} \ \ t_{\text {Hyp1}} = \frac{L_{\text {Hyp1}}}{\hat{\sigma }_{L_{\text {Hyp1}}}} \approx \frac{0.189}{0.025} \approx 7.55. \end{aligned}$$

The empirical *t*-value can then be compared to the critical *t*-value from a *t*-distribution with $N-K$ degrees of freedom and a predefined $\alpha$-level. For $\alpha =.05$ and a one-tailed test, the critical value is $t_{\text {crit}} = 1.65$. As $1.65 < 7.55$, we reject the null hypothesis. This result indicates that the contrast for Hypothesis 1, $\boldsymbol{\lambda }_{\text {Hyp1}} = (1.0, -0.5,-0.5)$, positively covaries with the observed mean pattern.

#### Example of a competing-contrast analysis for independent samples

Performing a competing-contrast analysis works analogously to the general procedure of contrast analysis described above. However, for competing-contrast analysis the standardized difference contrast weights are used.

18 $$\begin{aligned} L_{\text {difference}} \approx 0.19 \cdot 0.414 + (-0.71) \cdot 0.2 + 0.51 \cdot 0.25 \approx 0.064. \end{aligned}$$

Let us conduct a directional test assessing whether Hypothesis 1 aligns more closely to the observed data compared to Hypothesis 2. If both contrast vectors covary equally with the observed mean pattern or the contrast vector for Hypothesis 2 covaries stronger with the observed mean pattern, the contrast comparison value is $L \le 0$. If the contrast vector for Hypothesis 1 shows a stronger covariance with the observed mean pattern than Hypothesis 2, then $L > 0$.

To obtain the corresponding *t*-test statistic, we need to compute the standard error for the contrast estimate:

19 $$\begin{aligned} \hat{\sigma }_{L_{\text {difference}}}= &  \sqrt{\hat{\sigma }^2_{\text {within}}\sum _{i=1}^K \frac{\lambda ^2_i}{n_i}} \approx \sqrt{0.017\Big ( \frac{0.19^2}{40} + \frac{(-0.71)^2}{40} + \frac{0.51^2}{40} \Big )}\nonumber \\\approx &  \sqrt{0.017 \cdot 0.02} \approx 0.019, \end{aligned}$$

and thus

20 $$\begin{aligned} t_{\text {difference}} = \frac{L_{\text {difference}}}{\hat{\sigma }_{L_{\text {difference}}}} \approx \frac{0.064}{0.019} \approx 3.377. \end{aligned}$$

The resulting *t*-value is compared to the critical *t*-value from a *t*-distribution with $N-K$ degrees of freedom and a predefined $\alpha$-level of .05, which is $t_{\text {crit}} = 1.65$. We thus reject the null hypothesis of the competing-contrast analysis and conclude that the predicted mean pattern derived from Hypothesis 1 covaries more strongly with the observed mean pattern than the predicted mean pattern derived from Hypothesis 2. Using competing-contrast analysis, we were able to compare the two competing hypotheses using only one statistical test. In the original study, using a classic ANOVA approach, three post-hoc tests were necessary.

### Contrast analysis for independent samples in R 

In this section, we demonstrate how the cofad package can be used to perform contrast analyses in R. We assume that users are familiar with using R for statistical analyses. First, we show how to run a contrast analysis for independent samples using the data from the experiment by Maraver et al. (2021). The dataset is contained in the cofad package, and we encourage the reader to load the data and follow the steps using the accompanying R script.

We first load the cofad package and inspect the data.

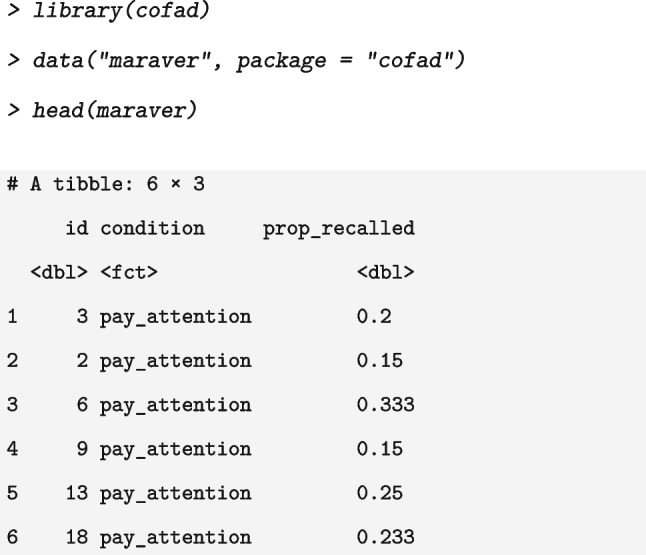



We see the data for several participants. The variable condition is a factor with three levels (*imagine*, *memorize*, *pay attention*). Each participant was assigned to one of these three conditions. The variable prop_recalled is the dependent variable. It gives the proportion of correctly recalled items for each participant. The hypothesis that we want to test is given by the following contrast vector $\boldsymbol{\lambda }_{\text {Hyp1}} = (1.0, -0.5, -0.5)$, as outlined above.

We can now call the calc_contrast function to run the analysis for the first contrast. The function takes four arguments: dv is used to specify the dependent variable, between is used to indicate the between-subjects factor, lambda_between is used to indicate the contrast weights for each level of the factor, and data is used to specify the dataset.

The output shows the contrast weights $\boldsymbol{\lambda }$ associated with each condition, the *t*-table, the *F*-table, and several measures of effect sizes. The *t*-table contains the contrast estimate *L*, the degrees of freedom (df) for the contrast, which is always 1, the *t*-value and the *p*-value. Note that the *p*-value is for a directional test, meaning the *t*-test is one-tailed.^4^ Directional hypotheses are frequently employed in contrast analyses, which is why this is the default setting in the cofad package. The *F*-table shows the sums of squares of the contrast (SS), the degrees of freedom for the contrast, the mean sums of squares, the *F*-value, and the non-directional *p*-value.

The output also shows several commonly used measures of effect size for contrast analysis, such as $r_{\text {effectsize}}$, $r_{\text {alerting}}$, and $r_{\text {contrast}}$ (Maxwell et al., 2004). Each effect size measure can be understood as the degree to which the observed mean pattern aligns with the predicted mean pattern. More precisely, $r_{\text {effectsize}}$ reflects the correlation between participants’ values on the dependent variable and the specified contrast weights. It is straightforward to calculate and interpret, and it provides a direct assessment of how well the participants’ values are described by the contrast. From an ANOVA perspective, $r_{\text {effectsize}}^2$ denotes the proportion of total variance that is explained by the contrast, similar in interpretation to the commonly used $\eta ^2$.

In addition, $r_{\text {alerting}}$ indicates the correlation between the observed group means and the contrast weights, and can be interpreted as a group-level correlation. In ANOVA terminology, $r_{\text {alerting}}^2$ corresponds to the proportion of between-group variance explained by the contrast weights. For instance, in datasets with high within-group variance that complicates the detection of group differences in a traditional omnibus ANOVA, a high $r_{\text {alerting}}$ can *alert* researchers that potentially meaningful differences in group means, aligned with the specified contrast, may be present. Importantly, $r_{\text {alerting}}$ serves as an upper boundary for $r_{\text {effectsize}}$.

Finally, $r_{\text {contrast}}$ represents the partial correlation between participants’ values on the dependent variable and the contrast weights, while partialing out the group differences not captured by the contrast. This measure is similar to $\eta _p^2$ in ANOVA. Although $r_{\text {contrast}}$ may be more complex to interpret than the other effect size measures in the R output, it has a direct connection to the significance test, making it valuable for power analysis. Researchers can use $r_{\text {contrast}}$ in power calculations and sample size planning, for example using the pwr.r.test function in the pwr package (Champely, 2020) or comparable functions in the pwrss package (Bulus, 2023) or the WebPower package (Zhang & Mai, 2023) in R as we discuss further below. At the same time, $r_{\text {contrast}}$ can be valuable for meta-analyses, particularly in cases in which different experiments involve different numbers of factors, while $r_{\text {effectsize}}$ would not allow for such comparisons (Furr, 2004). For additional information on the effect size measures, we recommend the works by Rosenthal et al. (2000), Furr (2004), Haans (2018), Rosnow et al. (2000), and Sedlmeier and Renkewitz (2008). Power analysis for contrasts using G*Power Faul et al. (2007, 2009) is discussed by Perugini et al. (2018).

In line with the results that we calculated by hand, the results from the cofad package suggest that the contrast for Hypothesis 1 describes the observed mean pattern in the data well. The *imagine* condition showed the highest memory performance compared to the average of the *memorize* condition and the *pay attention* conditions. Regarding the effect sizes, we can conclude that the contrast reflects the condition means closely (r_alerting), but the correlation between participants’ values on the dependent variable and the contrast weights (r_effectsize) is much smaller. The two effect size measures, hence, indicate that there remains unexplained interindividual heterogeneity within the experimental conditions.

### Competing-contrast analysis for independent samples in R 

We now demonstrate how competing-contrast analysis can be conducted using the cofad package. This approach allows us to directly compare Hypothesis 1 to Hypothesis 2. To test whether Hypothesis 1 ($\mu _{\textit{imagine} } > \mu _{\textit{memorize} } = \mu _{\textit{pay attention} }$) aligns more closely with the observed data in comparison to Hypothesis 2 ($\mu _{\textit{imagine} }> \mu _{\textit{memorize} } > \mu _{\textit{pay attention} }$), we first compute the standardized difference between the two contrast vectors. For this purpose, we can use the lambda_diff function in the cofad package. The researcher passes the two contrast vectors and a vector containing the labels of the factor levels. The function then computes the standardized difference between the contrast vectors, where lambda_favored is the contrast vector reflecting the favored hypothesis and lambda_rival is the contrast vector reflecting the rival hypothesis.

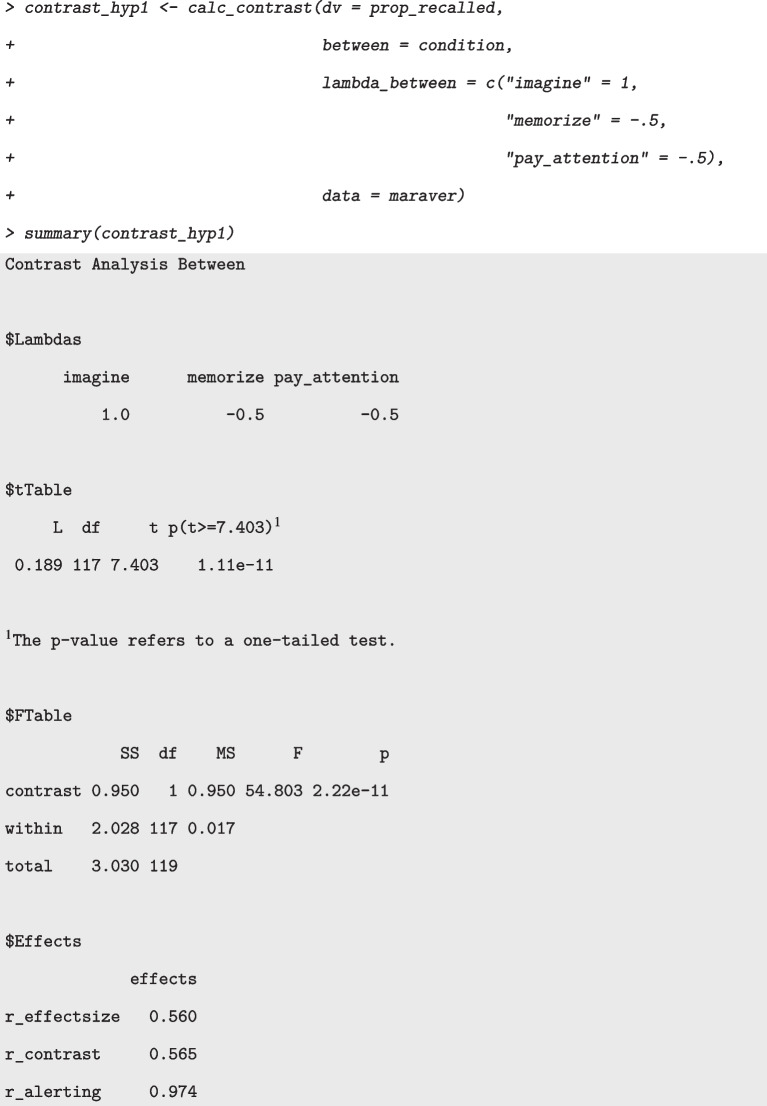



We can now use the standardized difference in contrast weights as a new contrast vector in the calc_contrast function from the cofad package:

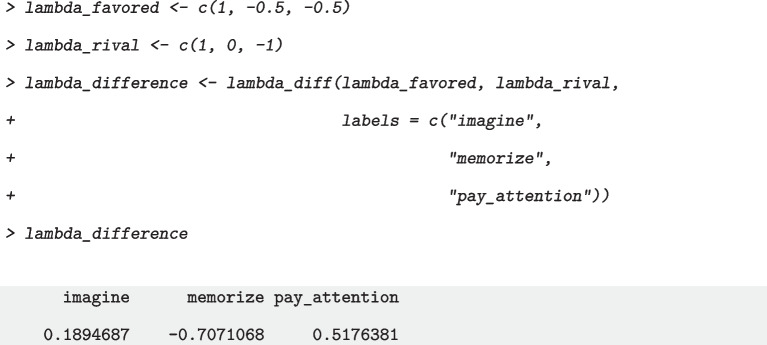



The results show that $p<\alpha$ and that all effect size measures deviate substantially from zero. Hence, we reject the null hypothesis and conclude that the predicted mean pattern derived from Hypothesis 1 covaries more strongly with the observed mean pattern in the data compared to the predicted mean pattern derived from Hypothesis 2. The effect size $r_{\text {alerting}}$ describes the correlation between the group means and the contrast weights of the difference contrast vector. The effect size $r_{\text {effectsize}}$ quantifies how strongly the predicted mean pattern based on Hypothesis 1 correlates with the participants’ observed values on the dependent variable when compared to Hypothesis 2. The effect sizes thus quantify the strength of the relationship between the observed data and the theory-based differences between the two hypotheses.

## Contrast analysis for dependent samples

Contrast analysis and competing-contrast analysis can also be used for dependent samples (within-subjects designs). Data from dependent samples are typically obtained in experiments with repeated measurements. Examples include testing the same participants at multiple time points (e.g., before a treatment, directly after a treatment, and a follow-up) or across multiple conditions (e.g., shallow, moderate, and deep encoding, or positive, negative, and neutral stimuli). The resulting measurements within one participant are not independent, which the statistical test needs to take into account.

When applied to dependent samples instead of independent samples, the main principle of contrast analysis remains the same. However, the test statistic and effect size measures are calculated differently. To illustrate the method of contrast analysis for dependent samples, we selected data from an experiment by Akan et al. (2018, Experiment 2B). The authors were interested in whether taking a memory test during the learning phase can improve or harm memory for contextual information.

**Fig. 2:**
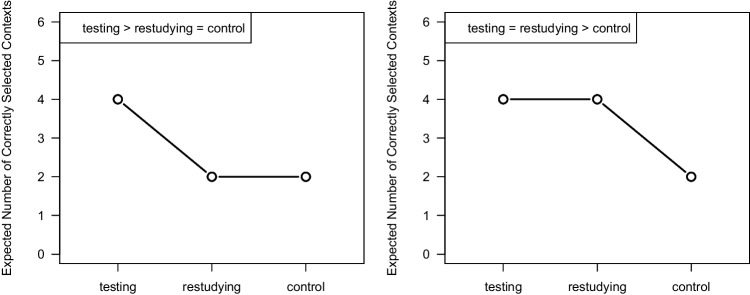
Visualization of the expected number of correctly selected contexts in the experiment by Akan et al. (2018). Note. *Left panel*: Visualization of Hypothesis 1, according to which *testing* leads to better context memory. *Right panel*: Visualization of Hypothesis 2, according to which *testing* does not lead to better context memory than *restudying*

The *testing effect* (also *retrieval-practice effect*) is defined as better memory performance after taking an initial test (i.e., practicing retrieval) compared to restudying the same material (Roediger & Karpicke, 2006). One popular explanation of the testing effect is the episodic-context account (Karpicke et al., 2014), which assumes that contextual information from the initial test is encoded alongside the central to-be-learned information. The contextual information may later act as retrieval cues and help to retrieve the central information during the final test. The original episodic-context account states that the relevant contextual information is temporal, whereas a more recent interpretation states that it can also be environmental (e.g., background scenes; Schwoebel et al., 2018).

To decide whether the episodic-context account can be extended from temporal to environmental cues, Akan et al. (2018) asked participants to study cue–target word pairs presented across different screen locations. The within-subjects factor was the type of practice each word pair received before the final test: cued-recall testing of the target given the cue (*testing* ), additional presentation (*restudying* ), and no additional exposure (*control*). The final test two days later included a cued-recall test (item test), followed by an alternative forced-choice test of the original item location (context test) that will be the focus of our reanalysis.

In the experiment by Akan et al. (2018), context memory measured as the proportion of correctly selected contexts was significantly higher for tested than restudied pairs, significantly higher for tested than control pairs, and did not differ significantly between restudied and control pairs. These results required three pairwise comparisons. However, the hypotheses can also be examined in a single test using competing-contrast analysis.

The hypothesis favored by Akan et al. (2018) and its rival hypothesis can be expressed using contrast weights for the three experimental conditions. For Hypothesis 1 ($\mu _{\textit{testing} } > \mu _{\textit{restudying} } = \mu _{\textit{control} }$), the contrast vector is $\boldsymbol{\lambda }_{\text {Hyp1}} = (1, -0.5, -0.5)$, which compares the *testing* condition to the average of *restudying* and *control* conditions. Alternatively, Hypothesis 2 ($\mu _{\textit{testing} } = \mu _{\textit{restudying} } > \mu _{\textit{control} }$) can be represented by the contrast vector $\boldsymbol{\lambda }_{\text {Hyp2}} = (0.5, 0.5, -1)$, contrasting the *control* condition with the average of the *testing* and *restudying* conditions. Figure 2 illustrates the predicted mean patterns for the two competing hypotheses, and Table 4 summarizes their contrast weights.

**Table 4:** Contrast weights specified for Hypothesis 1 and Hypothesis 2 of the experiment by Akan et al. (2018)

	*testing*	*restudying*	*control*
	$\lambda _1$	$\lambda _2$	$\lambda _3$
Hypothesis 1: $\mu _{\textit{testing} } > \mu _{\textit{restudying} } = \mu _{\textit{control} }$	1	$-0.5$	$-0.5$
Hypothesis 2: $\mu _{\textit{testing} } = \mu _{\textit{restudying} } > \mu _{\textit{control} }$	0.5	0.5	$-1$

### Statistical tests

In contrast analysis for dependent samples, the *L*-statistic reflects a measure of association between the contrast weights and the observed values of each person on the dependent variable for the different within-subjects conditions.

For $j \in (1,...,J)$ measurements (i.e., experimental conditions) and $i \in (1,...,N)$ participants, $L_i$ for person *i* is defined as:

21 $$\begin{aligned} L_i = \sum _{j = 1}^J(\lambda _j x_{ij}). \end{aligned}$$

To obtain the test statistic, $L_i$ is calculated for all participants in the dataset. Then, the average *L* across participants is taken to derive the contrast estimate $\bar{L}$:

22 $$\begin{aligned} \bar{L} = \frac{\sum _{i=1}^N (L_i)}{N}. \end{aligned}$$

Under the assumption that all participants provide data for all measurements (i.e., a balanced design, which is most often the case in repeated measures designs), one can test whether the contrast estimate $\bar{L}$ is significantly larger than zero using a *t*-test with $N-1$ degrees of freedom. The test statistic is calculated as follows:

23 $$\begin{aligned} t = \frac{\bar{L}}{\sqrt{\frac{\hat{\sigma }_L^2}{N}}} \end{aligned}$$

Note that $\hat{\sigma }_L^2$ is calculated as the sample variance of the $L_i$ values and provides an estimate for the population variance:^5^

24 $$\begin{aligned} \hat{\sigma }_L^2 = \frac{\sum _{i=1}^{N} (L_i - \bar{L})^2}{N-1}. \end{aligned}$$

#### Example of contrast analysis for dependent samples

We illustrate the procedure of contrast analysis for dependent samples using the data from the three conditions(*testing*, *restudying*, and *control*) in Experiment 2B reported by Akan et al. (2018). For illustrative purposes, we only calculate $\bar{L}$ and the *t*-statistic for the first four participants and Hypothesis 1 (but of course these statistics will need to be calculated for all participants as is done using R further below).

$L_{\text {Hyp1}}$ for Participant 1 is calculated as follows:

25 $$\begin{aligned} L_{\text {Hyp1}} = 1 \cdot 1 + (-0.5) \cdot 5 + (-0.5) \cdot 3 = -3. \end{aligned}$$

The resulting $L_{\text {Hyp1}}$ values for the four participants are shown in Table 5 together with the average $\bar{L}$ values calculated across the four participants. From the *L* values, the estimate of the population variance $\hat{\sigma }^2_{L}$ can be computed.

**Table 5:** Values of the dependent variable and $L_{\text {Hyp1}}$ for four participants and corresponding $\bar{L}$ and $\hat{\sigma }^2_{L}$ for Hypothesis 1

	*testing*	*restudying*	*control*	$L_{\text {Hyp1}}$
Participant 1	1	5	3	$-3$
Participant 2	1	4	3	$-2.5$
Participant 3	3	2	1	1.5
Participant 4	4	7	5	$-2$
$\bar{L}$				$-1.5$
$\hat{\sigma }^2_{L}$				4.17

Using $\bar{L}$ and $\hat{\sigma }^2_{L}$, we can then calculate the *t*-value of the four participants. The test statistic $t_{\text {Hyp1}}$ for these four participants is given by:

26 $$\begin{aligned} t_{\text {Hyp1}} = \frac{\bar{L}_{\text {Hyp1}}}{\sqrt{\frac{\hat{\sigma }^2_{L_{\text {Hyp1}}}}{N}}} = \frac{-1.5}{\sqrt{\frac{4.17}{4}}} = -1.47. \end{aligned}$$

This observed *t*-value can then be compared to the critical *t*-value from a *t*-distribution with $N-1$ degrees of freedom and a predefined $\alpha$-level. For $\alpha =.05$, we obtain $t_{\text {crit}}(3) = 2.35$ and retain the null hypothesis. Next, we conduct the contrast analysis using the cofad package, but this time for all 90 participants.

### Contrast analysis for dependent samples in R 

The R syntax for dependent sample contrast analysis in the cofad package is similar to the R syntax for independent samples. However, the data are structured in a different format because the data set contains multiple observations per participant. First, we load the data from Experiment 2B by Akan et al. (2018) with $N=90$ participants providing values in each of the three experimental conditions (*testing*, *restudying*, *control*).

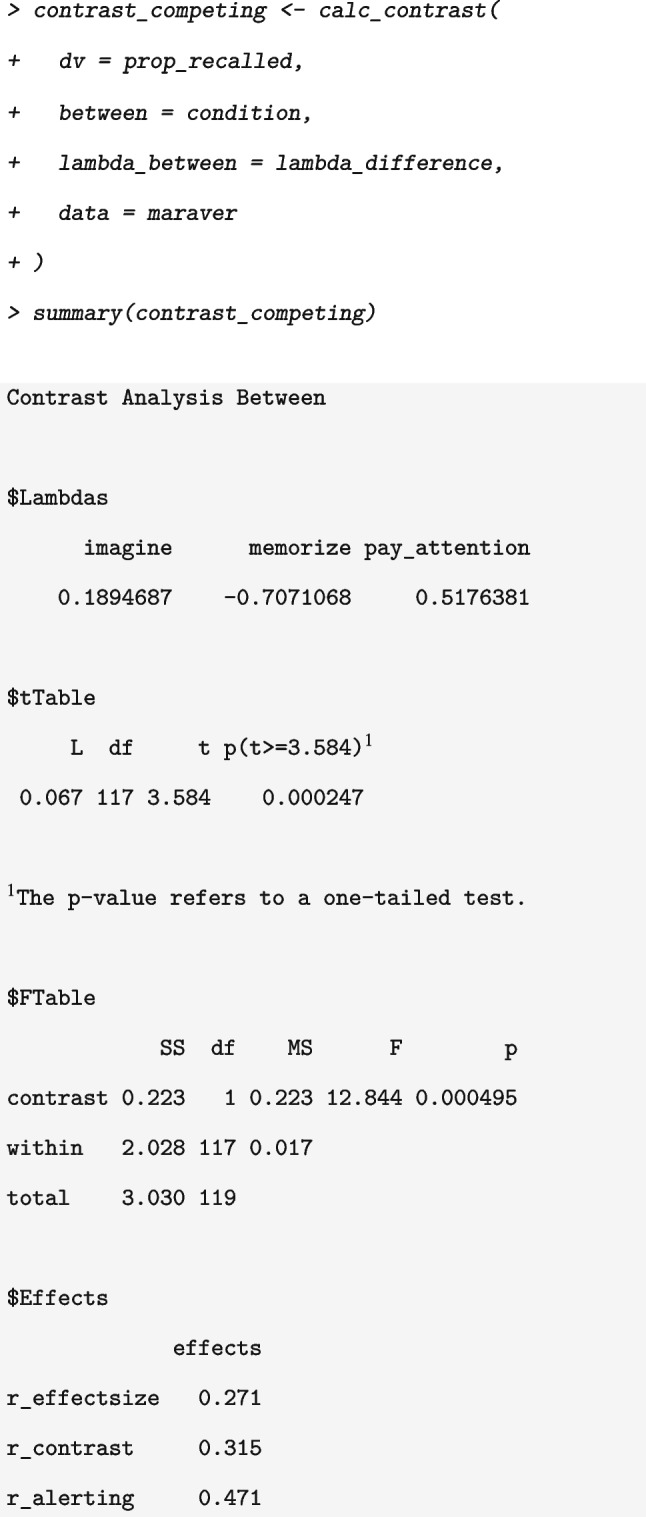



The dataset shows the participant id (subject), the within-subjects condition (condition), and the number of correctly selected contexts of each participant (contexts). This data format is also called the *long* format (as opposed to the wide format).

To conduct the contrast analysis for the dependent sample, we again apply the calc_contrast function from the cofad package, but this time with within, lambda_within, and id as arguments. The latter indicates each participant. We use the calc_contrast function to test the contrast reflecting Hypothesis 1 with the contrast vector $\boldsymbol{\lambda }_{\text {Hyp1}} = (1, -0.5, -0.5)$.

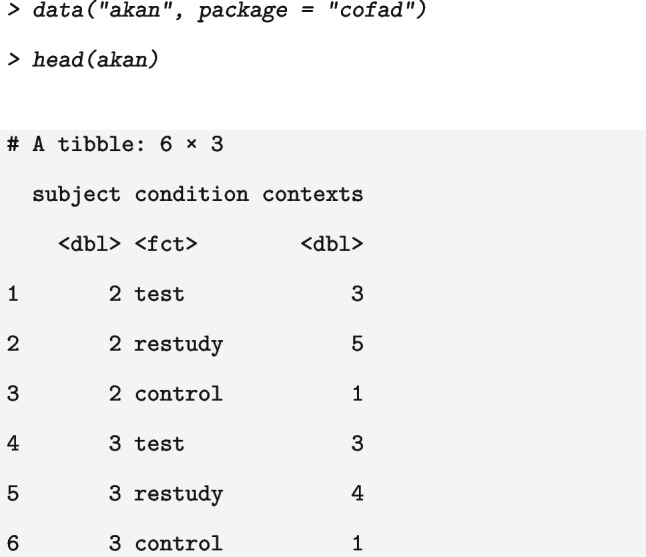



In the resulting output, the contrast estimate, standard error, degrees of freedom (df $=N-1),\,t$-value, and $p$-value are shown in the $t$-table. For within-subjects designs, the effect sizes $r_\text {contrast} { and}g_\text {contrast}$ are reported. The latter demonstrates how well the relative differences in the observed means between the conditions are reflected by the contrast weights, and it can serve as an estimate of Cohen’s $d$ in the population. For more details on effect sizes for dependent samples, we refer the interested reader to Rosenthal et al. (2000), Sedlmeier and Renkewitz (2008), Steiger (2004), and Wiens and Nilsson (2017).

The results in the R output suggest that the contrast for Hypothesis 1 describes the observed mean pattern well. In the *testing* condition, the number of correctly selected contexts seems to be higher compared to the average of the *restudying* and *control* conditions.

### Competing-contrast analysis for dependent samples in R 

We now conduct a competing-contrast analysis to test Hypothesis 1 against Hypothesis 2 (see Fig. 2 for the predicted mean patterns of the two hypotheses). Following the case of independent samples, we first calculate the standardized differences between both vectors of contrast weights.

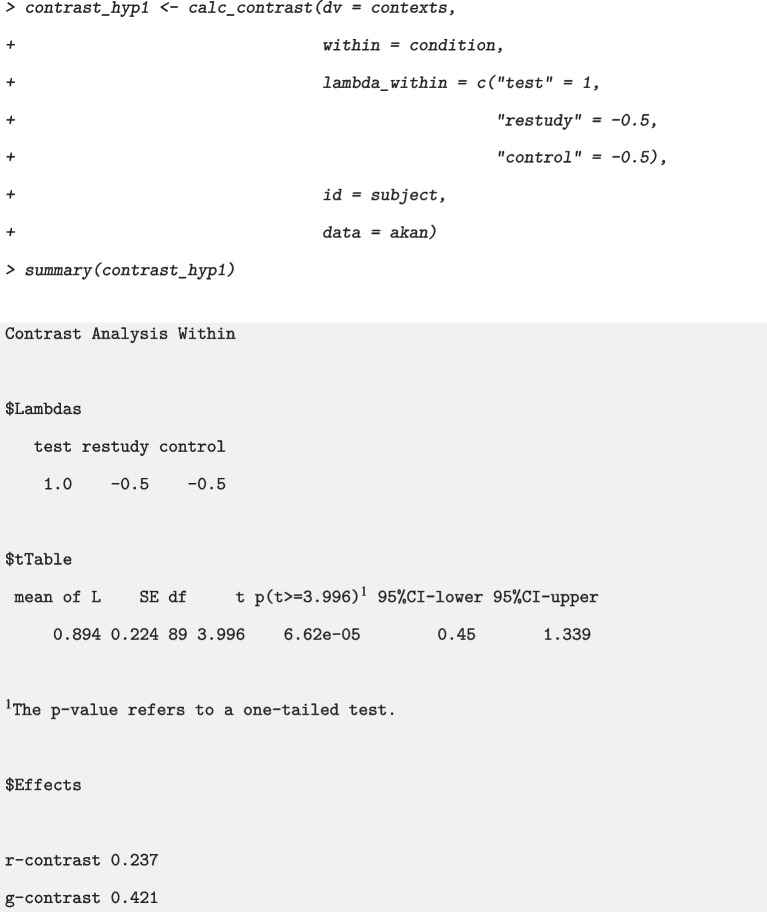



We can now conduct the competing-contrast analysis for dependent samples using the vector of contrast weight differences:

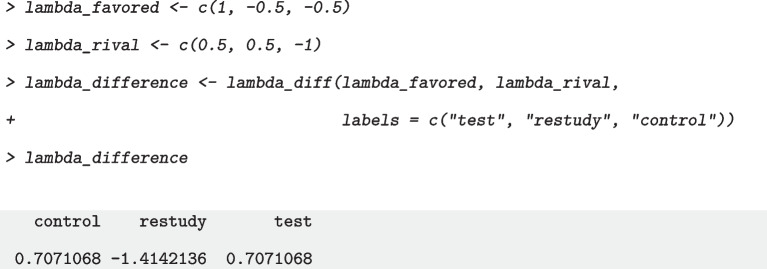



As for independent samples, the default *t*-test in the cofad package is directional such that a positive value of the *t*-statistic indicates that the favored contrast vector $\boldsymbol{\lambda } = (1, -0.5, -0.5)$ covaries more strongly with the observed mean pattern compared to the rival contrast vector $\boldsymbol{\lambda } = (0.5, 0.5, -1)$. The *t*-test is not significant and we retain the null hypothesis. Note that Akan et al. (2018) reached a similar conclusion, but had to run three *t*-tests.

## Power calculation in contrast analysis

For between-subjects designs, sample size planning and power analysis can be conducted using the effect size measure $r_\text {contrast}$ in combination with one of the many available power analysis packages in R. For example, using the pwr package, the required sample size for a between-subjects contrast analysis based on a *t*-test can be obtained by using the pwr.r.test function as follows:

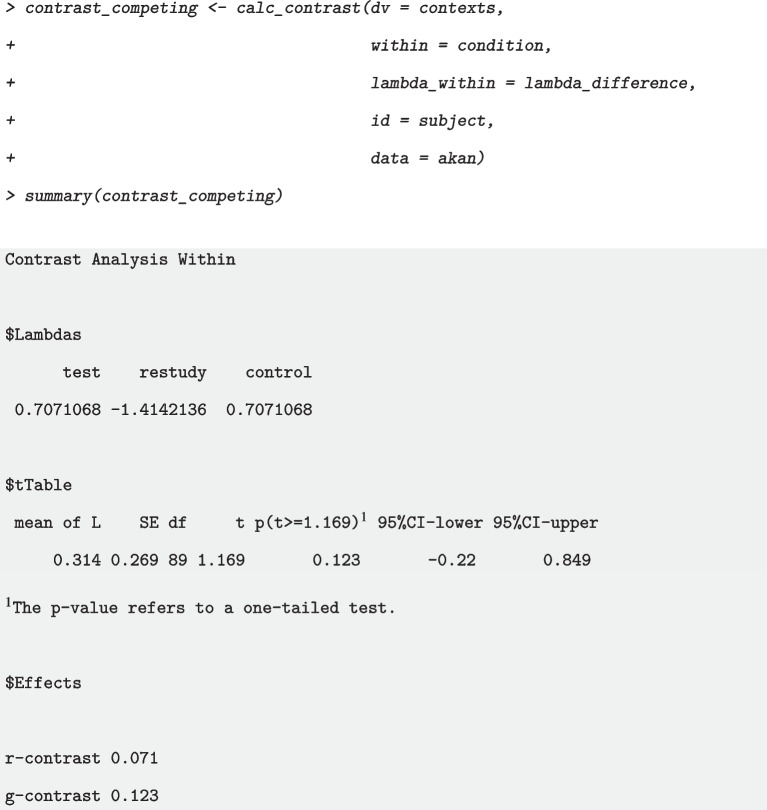



To achieve a minimum power of $80\%$ in the case of four conditions and a balanced design, 32 participants in total (due to rounding up) and eight participants per condition would be required. For the same parameters, Perugini et al. (2018) uses the non-directional *F*-test for contrast analysis, resulting in a recommendation to test nine participants per condition. Hence, as expected, a directional test is more powerful. This power calculation shows that contrast analysis allows researchers to test the predicted mean pattern in an efficient way. If the predicted mean pattern closely aligns with the observed mean pattern, as is the case in this example with an effect size of $r =.45$, a higher statistical power is achieved using contrast analysis compared to an omnibus ANOVA or post-hoc tests, which reduces the required sample size.

For within-subjects designs, the power analysis is based on a dependent-samples *t*-test. The output of cofad provides $g_\text {contrast}$, which can serve as an estimate of *d* in the population. In turn, *d* can be used for power analysis using the pwr.t.test function as follows:

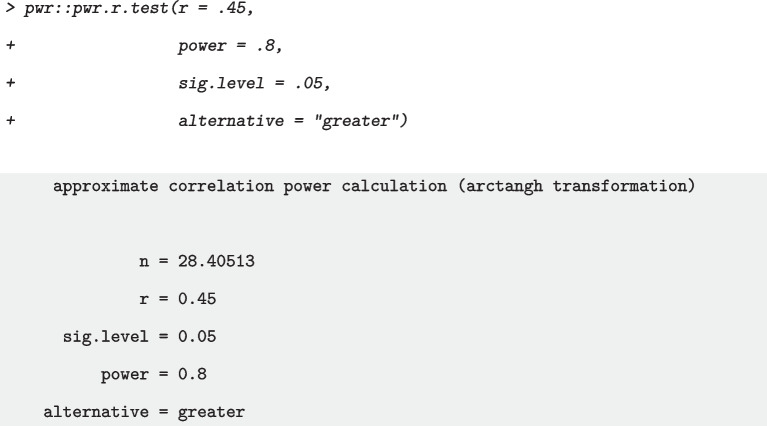



The power analysis results in a required sample size of $N=19$ participants to detect an effect of size $d = 0.8$ with a power of .95 in a within-subjects experiment. As with between-subjects designs, the *t*-test for dependent samples requires fewer participants when conducted as a one-tailed test.

As in power analyses for ANOVAs and other traditional statistical procedures, the technical aspects of power analysis for contrast analysis and competing-contrast analysis are relatively straightforward. However, selecting theoretically meaningful estimates for the effect size measure $r_\text {contrast}$ remains a key challenge.

Because $r_\text {effectsize}$ is easier to estimate, one possibility is to assume that $r_\text {alerting}$ is equal to one, which implies $r_\text {contrast} = r_\text {effectsize}$. In case that $r_\text {alerting}$ is smaller than one in reality, $r_\text {effectsize}$ serves as a lower bound for the true effect size and the power analysis will be conservative. Another possibility is to estimate $r_\text {contrast}$ directly, which requires knowing the sums of squares of the contrast under investigation and the sums of squares within groups. The size of these properties can be determined using data from previous studies or by conducting a pilot study. Alternatively, one could use a simulation approach (see Strobl et al., 2024) to generate plausible simulated data and calculate $r_\text {contrast}$ from this data.

## Discussion

This tutorial article provided a tutorial to conduct contrast analysis using the R package cofad and outlined a rarely used method to compare two competing predictions via competing-contrast analysis. Contrast analysis offers a theory-driven, flexible, and powerful framework for testing directional, a priori hypotheses about mean patterns across experimental conditions or measurement points. Rather than relying on omnibus tests like ANOVA, contrast analysis allows researchers to directly evaluate the extent to which observed mean patterns align with predicted ones. Competing-contrast analysis extends this framework by enabling direct comparisons between two predicted patterns of condition means derived from competing theories, offering a structured statistical approach to theory testing.

A central element of contrast analysis is the specification of contrast weights, which translate theoretical predictions into testable statistical hypotheses (see Abelson & Prentice, 1997; Rosenthal et al., 2000; Rosnow & Rosenthal, 1996; Furr, 2008). For researchers unfamiliar with contrast analysis, deriving these weights may seem daunting at first. However, as demonstrated in our practical examples, this process often clarifies theoretical expectations and the interpretation of study results, and is thus a valuable step in the research process that deserves more attention. Pre-registering both the predicted mean patterns and the corresponding contrast weights can improve transparency, prevent post-hoc adjustments, and help maintain the confirmatory nature of hypothesis testing (e.g., Lakens, 2019; Maier & Lakens, 2022; Francis, 2012b; Francis, 2012a, for discussions). At the same time, the utility of pre-registration depends on how well the contrasts are theoretically derived. Reviewers should therefore examine whether the contrast weights are grounded in theoretical considerations.

While standard contrast analysis evaluates the degree to which a predicted pattern fits the data, competing-contrast analysis serves a different purpose: It assesses which of two competing, theory-driven predictions is more strongly supported by the observed results. This approach is especially valuable when different theoretical accounts lead to different expectations about mean patterns across conditions. In such cases, the research question shifts from asking whether a particular pattern fits to asking which of two competing hypotheses provides a better account of the data. Importantly, competing-contrast analysis does not test the absolute fit of either prediction, but rather their relative alignment with the observed pattern.

The fact that opposing hypotheses can be compared may make competing-contrast analysis a suitable statistical complement to so-called *experimentum crucis* designs in psychology. In the tradition of experimental science, an experimentum crucis can be understood as a critical experiment that favors one hypothesis and rules out the other (Platt, 1964; Lohne, 1968; Malejka et al., 2022; Sinico, 2018). Although psychological theories are often underspecified and empirical data tend to be noisy, researchers can often derive distinct predictions from competing theories. Competing-contrast analysis may thus offer a statistically rigorous way to evaluate such predictions, making it a promising tool in contexts in which theory comparison is the primary objective.

Another consideration when applying contrast analysis, especially when testing multiple contrasts as part of a contrast set, is how to handle the issue of multiple testing. The literature remains divided on whether adjustments to the significance level $\alpha$ are required when conducting multiple hypothesis tests (e.g., Bennett et al., 2009; O’Keefe, 2003; Sinclair et al., 2013; Wilson, 1962). Rubin (2021) and Weber (2007) have highlighted that the type of multiple testing matters, that is, whether the goal is to evaluate a disjunction (e.g., at least one effect is present), a conjunction (e.g., all effects must be present), or whether individual hypotheses are tested independently. In contrast analysis, these considerations may imply that multiple individual directional hypotheses (e.g., $\mu _1 > \text {mean}(\mu _2, \mu _3)$ and $\mu _2 = \mu _3$) can be tested separately without necessarily requiring adjustments to the significance level $\alpha$ – provided that each hypothesis is interpreted independently and not as part of a single, overarching hypothesis about the entire mean pattern. Because competing-contrast analysis involves a single, planned comparison between two competing predictions, it constitutes a planned test of one hypothesis and thus does not raise concerns about inflated type-I error rates due to multiple testing.

Despite its advantages, contrast analysis remains underutilized in published psychological research. This is somewhat surprising given that it is widely taught as part of psychological study programs. A recent survey of professors teaching methods and statistics at German universities found that 28 out of 34 B.Sc. and M.Sc. psychology programs teach contrast analysis (Sternkopf et al., 2025), suggesting a gap between educational standards and research practices. We believe that user-friendly R packages, accessible code scripts, and hands-on tutorials can help bridge this gap. In addition to the functions presented in this tutorial, the cofad package includes an integrated Shiny app that allows users to interactively select variables and define contrast weights. While scripted code is generally preferable for reproducibility and sharing, the app’s graphical interface may serve as a useful teaching aid in statistics education.

In sum, contrast analysis provides a more targeted and theoretically informative alternative to omnibus ANOVAs, particularly when researchers have clear predictions about expected patterns. In the light of the replication crisis and calls to increase statistical power in psychology, adopting contrast analysis for confirmatory research is a valuable approach. As an extension to standard contrast analysis, competing-contrast analysis adds further value by enabling researchers to directly test two competing hypotheses against each other, supporting stronger, theory-based inferences. In the future, we hope that contrast analysis, and in particular competing-contrast analysis, will become a standard method in the statistical toolbox of psychological researchers.

## Data Availability

Datasets are available in the cofad package (https://cran.r-project.org/web/packages/cofad/index.html; Titz and Burkhardt, 2021; Titz and Burkhardt, 2024).

## Appendix: Typical contrast vectors and orthogonal contrasts

### Contrast vectors representing ANOVA main and interaction effects

In factorial designs, such as a $2 \times 2$ factorial ANOVA, traditional main and interaction effects can be represented by a set of orthogonal contrasts. These contrasts are mathematically equivalent to the effects estimated in an ANOVA and offer the additional benefit of allowing for directional, hypothesis-driven testing. Table 6 shows the set of orthogonal contrasts for main and interaction effects in a $2 \times 2$ factorial design with four conditions. The first contrast represents the main effect of Factor A, the second contrast represents the main effect of Factor B, and the third contrast represents the interaction between the two factors.

**Table 6:** Contrast vectors representing main and interaction effects in a $2 \times 2$ factorial design ($A \times B$ with two factor levels each)

	A1		A2	
	B1	B2	B1	B2
Type of Effect	$\lambda _{1}$	$\lambda _{2}$	$\lambda _{3}$	$\lambda _{4}$
Main Effect of Factor A	$-1$	$-1$	1	1
Main Effect of Factor B	$-1$	1	$-1$	1
Interaction Effect	1	$-1$	$-1$	1

*Note:* A1, A2, B1, and B2 are factor levels of the two factors, and $\lambda _{i}$ are the contrast weights for each combination in the $2\times 2$ factorial design

#### Typical contrast vectors

There exists a number of commonly used contrast vectors. Examples include polynomial, Helmert, simple, and repeated contrasts (see Rosenthal et al., 2000; Draper & Smith, 1998; Saville & Wood, 1991). Table 7 shows a selection of these contrast vectors for designs with four groups or conditions. Given *K* experimental groups, researchers can specify a maximum $K-1$ contrasts (which corresponds to the numerator degrees of freedom in ANOVA).

**Table 7:** Selected examples of commonly used contrast vectors for four groups

Type of Contrast	Description		$\lambda _{1}$	$\lambda _{2}$	$\lambda _{3}$	$\lambda _{4}$	Orthogonal Set
Polynomial	linear, quadratic, and cubic trend	Contrast A	$-1$	$-\frac{1}{3}$	$\frac{1}{3}$	1	yes
		Contrast B	$\frac{1}{2}$	$-\frac{1}{2}$	$-\frac{1}{2}$	$\frac{1}{2}$	
		Contrast C	$-1$	$\frac{1}{3}$	$-\frac{1}{3}$	1	
Helmert	comparing each group with the following groups	Contrast A	1	$-\frac{1}{3}$	$-\frac{1}{3}$	$-\frac{1}{3}$	yes
		Contrast B	0	1	$-\frac{1}{2}$	$-\frac{1}{2}$	
		Contrast C	0	0	1	$-1$	
Simple	comparing each group with a reference (typically the last) group	Contrast A	1	0	0	$-1$	no
		Contrast B	0	1	0	$-1$	
		Contrast C	0	0	1	$-1$	
Repeated	comparing each group with the next group	Contrast A	1	$-1$	0	0	no
		Contrast B	0	1	$-1$	0	
		Contrast C	0	0	1	$-1$	

The rightmost column in Tables 6 and 7 indicates whether each pair of contrast vectors is orthogonal. Orthogonal contrasts have the attractive property that when the design is balanced (i.e., the group sizes are equal), they are completely independent from each other. Therefore, orthogonal contrasts address non-overlapping research questions. Hence, the sum of the variance of the dependent variable explained by the $K-1$ contrasts is equal to the variance explained by the between-group factor in an ANOVA when the groups have equal sample sizes (Rosenthal et al., 2000; Maxwell et al., 2004). When two contrasts are not orthogonal, or group sizes are not equal, the contrasts can be analyzed jointly in order to avoid capitalizing on the proportion of explained variance (see e.g., Kelley, 2007; Hothorn, Bretz, & Westfall, 2008; Lenth, 2016, for R packages).

Whether two contrasts are orthogonal can be determined by calculating the sum of the dot product of the contrast weights. When the sum is zero, the two contrasts are orthogonal:

27 $$\begin{aligned} \sum _{i=1}^K \lambda _{i,\text {A}}\lambda _{i,\text {B}} = 0. \end{aligned}$$

We illustrate the procedure of how to determine whether two contrasts are orthogonal using the example of Contrast A and Contrast B of the set of polynomial contrasts for four groups from Table 7:

**Table:**

	$\lambda _{1}$	$\lambda _{2}$	$\lambda _{3}$	$\lambda _{4}$
Contrast A	$-1$	$-\frac{1}{3}$	$\frac{1}{3}$	1
Contrast B	$\frac{1}{2}$	$-\frac{1}{2}$	$-\frac{1}{2}$	$\frac{1}{2}$

The contrast weights in each column are multiplied, and the resulting products are summed up:

28 $$\begin{aligned} \sum _{i=1}^K \lambda _{i,\text {A}}\lambda _{i,\text {B}}&= (-1) \cdot \frac{1}{2} + \big (-\frac{1}{3}\big ) \cdot \big (-\frac{1}{2}\big ) + \frac{1}{3} \cdot \big (-\frac{1}{2}\big ) + 1 \cdot \frac{1}{2}\nonumber \\&= -\frac{1}{2} + \frac{1}{6} - \frac{1}{6} + \frac{1}{2}\nonumber \\&= 0. \end{aligned}$$

Since the sum of the product of the contrast weights is 0, we can conclude that the contrasts are orthogonal.

Note that although orthogonal contrasts are preferred where possible, orthogonal contrasts add to the benefits of contrast analysis rather than being a requirement. Hence, when a priori hypotheses can be derived from theory, the contrast weights reflecting these hypotheses should be used even if they are non-orthogonal (Rosenthal et al., 2000).

## Footnotes

1 In this article, we use the term *condition* to describe (experimental) groups in between-subjects designs and measurement points in within-subjects designs.

2 In this case of orthogonal contrast vectors and equal group sizes, the sums of squares of the $K-1$ contrasts for *K* groups or conditions will exactly match the sums of squares of the ANOVA analysis (Haans, 2018; Rosenthal et al., 2000).

3 The expression $\sum _{i=1}^K \lambda _i \bar{x}_i$ can be understood as a simplified form of the covariance between the contrast weights and the group means, $\sum _{i=1}^K (\lambda _i - \bar{\lambda })(\bar{x}_i - \bar{\bar{x}})$. This quantity is proportional to the (unstandardized) covariance between the two vectors. Because the contrast weights are chosen to sum to zero $\bar{\lambda } = 0$, the expression simplifies to $\sum _{i=1}^K \lambda _i \bar{x}_i$.

4 Differences in the calculation of the contrast estimate and *t*-value compared to the above are due to rounding.

5 For unequal group sizes, the harmonic mean is used to estimate the pooled variance (e.g., Sedlmeier and Renkewitz, 2008).

## Code Availability

Code is available on OSF: https://osf.io/ny5b6.

## Declarations

### Author Note

We would like to thank Markus Burkhardt for insightful discussions and Sandro Menegola for preparing the dataset with independent samples.

## References

[CR1] Abelson, R. P., & Prentice, D. A. (1997). Contrast tests of interaction hypotheses. *Psychological Methods,**2*, 315–328. 10.1037/1082-989X.2.4.315

[CR2] Akan, M., Stanley, S. E., & Benjamin, A. S. (2018). Testing enhances memory for context. *Journal of Memory and Language,**103*, 19–27. 10.1016/j.jml.2018.07.003

[CR3] Bennett, C. M., Wolford, G. L., & Miller, M. B. (2009). The principled control of false positives in neuroimaging. *Social Cognitive and Affective Neuroscience,**4*(4), 417–422. 10.1093/scan/nsp053 20042432 10.1093/scan/nsp053 PMC2799957

[CR4] Bereiter, C., & Scardamalia, M. (1989). Intentional learning as a goal of instruction. In L. B. Resnick (Ed.), *Knowing, learning, and instruction: Essays in honor of Robert Glaser* (pp. 361–392). Erlbaum.

[CR5] Bernstein, D. M., Scoboria, A., Desjarlais, L., & Soucie, K. (2018). “False memory’’ is a linguistic convenience. *Psychology of Consciousness: Theory, Research, and Practice,**5*(2), 161–179. 10.1037/cns0000148

[CR6] Brewer, W. F. (1977). Memory for the pragmatic implications of sentences. *Memory & Cognition,**5*(6), 673–678. 10.3758/BF03197414 24203284 10.3758/BF03197414

[CR7] Bulus, M. (2023). pwrss: Statistical power and sample size calculation tools [R package version 0.3.1]. https://CRAN.R-project.org/package=pwrss

[CR8] Champely, S. (2020). pwr: Basic functions for power analysis [R package version 1.3-0]. https://CRAN.R-project.org/package=pwr

[CR9] Craik, F. I., & Lockhart, R. S. (1972). Levels of processing: A framework for memory research. *Journal of Verbal Learning and Verbal Behavior,**11*(6), 671–684. 10.1016/S0022-5371(72)80001-x

[CR10] de Melo, C. M., & Terada, K. (2020). The interplay of emotion expressions and strategy in promoting cooperation in the iterated prisoner’s dilemma. *Scientific Reports,**10*, 1–8. 10.1038/s41598-020-71919-6 32917943 10.1038/s41598-020-71919-6 PMC7486426

[CR11] Draper, N. R., & Smith, H. (1998). *Applied regression analysis*. Wiley Series in Probability and Statistics. 10.1002/9781118625590

[CR12] Faul, F., Erdfelder, E., Buchner, A., & Lang, A. G. (2009). Statistical power analyses using G*Power 3.1: Tests for correlation and regression analyses. *Behavior Research Methods,**41*(4), 1149–1160. 10.3758/BRM.41.4.1149 19897823 10.3758/BRM.41.4.1149

[CR13] Faul, F., Erdfelder, E., Lang, A.-G., & Buchner, A. (2007). G*Power 3: A flexible statistical power analysis program for the social, behavioral, and biomedical sciences. *Behavior Research Methods,**39*(2), 175–191. 10.3758/BF03193146 17695343 10.3758/bf03193146

[CR14] Fitts, D. A. (2010). The variable-criteria sequential stopping rule: Generality to unequal sample sizes, unequal variances, or to large ANOVAs. *Behavior Research Methods,**42*(4), 918–929. 10.3758/BRM.42.4.918 21139159 10.3758/BRM.42.4.918

[CR15] Foley, M. A., Wozniak, K. H., & Gillum, A. (2006). Imagination and false memory inductions: Investigating the role of process, content and source of imaginations. *Applied Cognitive Psychology,**20*(9), 1119–1141. 10.1002/acp.1265

[CR16] Francis, G. (2012a). Publication bias and the failure of replication in experimental psychology. *Psychonomic Bulletin & Review,**19*(6), 975–991. 10.3758/s13423-012-0322-y 23055145 10.3758/s13423-012-0322-y

[CR17] Francis, G. (2012b). Too good to be true: Publication bias in two prominent studies from experimental psychology. *Psychonomic Bulletin & Review,**19*(2), 151–156. 10.3758/s13423-012-0227-9 22351589 10.3758/s13423-012-0227-9

[CR18] Furr, R. M. (2004). Interpreting effect sizes in contrast analysis. *Understanding Statistics,**3*, 1–25. 10.1207/s15328031us0301_1

[CR19] Furr, R. M. (2008). A contrast analysis approach to change. *Educational Research and Evaluation,**14*(4), 335–362. 10.1080/13803610802249571

[CR20] Haans, A. (2018). Contrast analysis: A tutorial. *Practical Assessment, Research and Evaluation,**23*(9), 1–21. 10.7275/7dey-zd62

[CR21] Hothorn, T., Bretz, F., & Westfall, P. (2008). Simultaneous inference in general parametric models. *Biometrical Journal,**50*(3), 346–363. 10.1002/bimj.200810425 18481363 10.1002/bimj.200810425

[CR22] Kaltenbach, H.-M. (2021). Comparing treatment groups with linear contrasts. In H.-M. Kaltenbach (Ed.), *Statistical design and analysis of biological experiments* (pp. 97–120). Springer International Publishing. 10.1007/978-3-030-69641-2_5

[CR23] Karpicke, J. D., Lehman, M., & Aue, W. R. (2014). Retrieval-based practice: An episodic context account. In B. H. Ross (Ed.), *Psychology of learning and motivation* (pp. 237–284). Elsevier Academic Press. 10.1016/b978-0-12-800283-4.00007-1

[CR24] Kelley, K. (2007). Methods for the behavioral, educational, and social sciences: An R package. *Behavior Research Methods,**39*(4), 979–984. 10.3758/BF03192993 18183915 10.3758/bf03192993

[CR25] Lachner, A., Backfisch, I., Hoogerheide, V., van Gog, T., & Renkl, A. (2019). Timing matters! Explaining between study phases enhances students’ learning. *Journal of Educational Psychology,**112*, 841–853. 10.1037/edu0000396

[CR26] Lakens, D. (2019). The value of preregistration for psychological science: A conceptual analysis. *Japanese Psychological Review,**62*(3), 221–230. 10.24602/sjpr.62.3_221

[CR27] Langenberg, B., Janczyk, M., Koob, V., Kliegl, R., & Mayer, A. (2023). A tutorial on using the paired t test for power calculations in repeated measures ANOVA with interactions. *Behavior Research Methods,**55*(5), 2467–2484. 10.3758/s13428-022-01902-8 36002625 10.3758/s13428-022-01902-8 PMC10439102

[CR28] Lenth, R. V. (2016). Least-squares means: The R package lsmeans. *Journal of Statistical Software, **69*(1). 10.18637/jss.v069.i01

[CR29] Lohne, J. A. (1968). Experimentum crucis. *Notes and Records of the Royal Society of London, **23*(2), 169–199. https://www.jstor.org/stable/530985

[CR30] Maier, M., & Lakens, D. (2022). Justify your alpha: A primer on two practical approaches. *Advances in Methods and Practices in Psychological Science,**5*(2), 25152459221080396. 10.1177/25152459221080396

[CR31] Malejka, S., Heck, D. W., & Erdfelder, E. (2022). Recognition-memory models and ranking tasks: The importance of auxiliary assumptions for tests of the two-high-threshold model. *Journal of Memory and Language,**127*, 104356 . 10.1016/j.jml.2022.104356

[CR32] Maraver, M. J., Lapa, A., Garcia-Marques, L., Carneiro, P., & Raposo, A. (2021). Imagination reduces false memories for everyday action sentences: Evidence from pragmatic inferences. *Frontiers in Psychology,**12*, Article 668899. 10.3389/fpsyg.2021.668899 34489789 10.3389/fpsyg.2021.668899 PMC8417559

[CR33] Maxwell, S. E., Delaney, H. D., & Kelley, K. (2004). *Designing experiments and analyzing data: A model comparison perspective* (2nd ed.). Routledge.

[CR34] O’Keefe, D. J. (2003). Colloquy: Should familywise alpha be ajusted? Against familywise alpha adjustment. *Human Communication Research,**29*(3), 431–447. 10.1111/j.1468-2958.2003.tb00846.x

[CR35] Perugini, M., Gallucci, M., & Costantini, G. (2018). A practical primer to power analysis for simple experimental designs. *International Review of Social Psychology,**31*, 1–23. 10.5334/IRSP.181

[CR36] Platt, J. R. (1964). Strong inference. *Science,**146*(3642), 347–353. 10.1126/science.146.3642.347 17739513 10.1126/science.146.3642.347

[CR37] Roediger, H. L., & Karpicke, J. D. (2006). The power of testing memory. *Perspectives on Psychological Science,**1*, 181–210. 10.1111/j.1745-6916.2006.00012.x 26151629 10.1111/j.1745-6916.2006.00012.x

[CR38] Rosenthal, R., & Rosnow, R. (1985). *Contrast analysis*. Cambridge University Press. Retrieved July 29, 2025, from https://www.cambridge.org/universitypress/subjects/psychology/psychologyresearch-methods-and-statistics/contrast-analysis-focused-comparisons-analysis-variance

[CR39] Rosenthal, R., Rosnow, R. L., & Rubin, D. B. (2000). *Contrasts and effect sizes in behavioral research*. Cambridge University Press. www.cambridge.org/9780521659802

[CR40] Rosnow, R. L., & Rosenthal, R. (1996). Contrasts and interactions redux: Five easy pieces. *Psychological Science,**7*(4), 253–258. 10.1111/j.1467-9280.1996.tb00369.x

[CR41] Rosnow, R. L., Rosenthal, R., & Rubin, D. B. (2000). Contrasts and correlations in effect-size estimation. *Psychological Science,**11*(6), 446–453. 10.1111/1467-9280.00287 11202488 10.1111/1467-9280.00287

[CR42] Rubin, M. (2021). When to adjust alpha during multiple testing: A consideration of disjunction, conjunction, and individual testing. *Synthese,**199*(3), 10969–11000. 10.1007/s11229-021-03276-4

[CR43] Saville, D. J., & Wood, G. R. (1991). *Statistical Methods: The Geometric Approach*. New York: Springer.

[CR44] Schwoebel, J., Depperman, A. K., & Scott, J. L. (2018). Distinct episodic contexts enhance retrieval-based learning. *Memory,**26*, 1291–1296. 10.1080/09658211.2018.1464190 29649927 10.1080/09658211.2018.1464190

[CR45] Sedlmeier, P., & Renkewitz, F. (2008). Kontrastanalyse. In *Forschungsmethoden und Statistik in der Psychologie* (pp. 511–541). Pearson Studium.

[CR46] Sinclair, J., Taylor, P., & Hobbs, S. (2013). Alpha level adjustments for multiple dependent variable analyses and their applicability - A review. *International Journal of Sports Science and Engineering,**7*(1), 17–20.

[CR47] Sinico, M. (2018). Why experimentum crucis is possible in psychology of perception. *Gestalt Theory,**40*(1), 45–57. 10.2478/gth-2018-0003

[CR48] Steiger, J. H. (2004). Beyond the F-test: Effect size confidence intervals and tests of close fit in the analysis of variance and contrast analysis. *Psychological Methods,**9*, 164–182. 10.1037/1082-989X.9.2.164 10.1037/1082-989X.9.2.164 15137887

[CR49] Sternkopf, A., Lungwitz, V. Zacke, T., & Titz, J. (2025). Einheitliche oder divers? Die Methoden Psychologiestudium in Deutschland [under review].

[CR50] Strobl, C., Henninger, M., Rothacher, Y., & Debelak, R. (2024). *Simulationsstudien in R: Design und praktische Durchführung*. Springer. 10.1007/978-3-662-70561-2

[CR51] Thapar, A., & McDermott, K. B. (2001). False recall and false recognition induced by presentation of associated words: Effects of retention interval and level of processing. *Memory & Cognition,**29*(3), 424–432. 10.3758/BF03196393 10.3758/bf03196393 11407419

[CR52] Titz, J., & Burkhardt, M. (2024). cofad: Contrast analyses for factorial designs. https://CRAN.R-project.org/package=cofad

[CR53] Titz, J., & Burkhardt, M. (2021). cofad: An R package and shiny app for contrast analysis. *Journal of Open Source Software,**6*(67), 3822. 10.21105/joss.03822

[CR54] Vorauer, J. D., Petsnik, C., & Quesnel, M. S. (2020). Who brings you up when you’re feeling down? Distinct implications of dispositional empathy versus situationally-prompted empathic mindsets for targets’ affective experience in face-to-face interpersonal interaction. *Journal of Experimental Social Psychology,**89*, 1–12. 10.1016/j.jesp.2020.103991

[CR55] Wahlsten, D. (1991). Sample size to detect a planned contrast and a one degree-of-freedom interaction effect. *Psychological Bulletin,**110*, 587–595. 10.1037/0033-2909.110.3.587

[CR56] Weber, R. (2007). Responses to Matsunaga: To adjust or not to adjust alpha in multiple testing: That is the question. Guidelines for alpha adjustment as response to O’Keefe’s and Matsunaga’s critiques. *Communication Methods and Measures,**1*(4), 281–289. 10.1080/19312450701641391

[CR57] Wiens, S., & Nilsson, M. E. (2017). Performing contrast analysis in factorial designs: From NHST to confidence intervals and beyond. *Educational and Psychological Measurement,**77*(4), 690–715. 10.1177/0013164416668950 29805179 10.1177/0013164416668950 PMC5952862

[CR58] Wilson, W. (1962). A note on the inconsistency inherent in the necessity to perform multiple comparisons. *Psychological Bulletin,**59*(4), 296–300. 10.1037/h0040447 14007431 10.1037/h0040447

[CR59] Zhang, Z., & Mai, Y. (2023). WebPower: Basic and advanced statistical power analysis [R package version 0.9.4]. https://CRAN.R-project.org/package=WebPower

